# Management of Osteogenesis Imperfecta Complicated by Severe Pneumonia in a Resource‐Limited Setting: A Case Report

**DOI:** 10.1155/crpe/2264928

**Published:** 2026-04-07

**Authors:** Bipesh Kumar Shah, Diwakar Koirala, Shilu Upreti, Megha Mahato, Bivek Mishra, Ramesh Sapkota, Dibya Raj Chaudhary

**Affiliations:** ^1^ Department of Pediatrics, B.P. Koirala Institute of Health Sciences, Dharan, Nepal, bpkihs.edu; ^2^ Nobel Medical College and Teaching Hospital, Biratnagar, Nepal, nobelmedicalcollege.com.np

**Keywords:** multidisciplinary care, osteogenesis imperfecta, pneumonia, resource-limited setting, respiratory complications

## Abstract

**Introduction:**

Osteogenesis imperfecta (OI) is a rare genetic disorder characterized by bone fragility, skeletal deformities, and multisystem involvement, with respiratory complications representing a major cause of morbidity and mortality. Intrinsic pulmonary abnormalities, restrictive physiology from scoliosis, and impaired secretion clearance increase susceptibility to respiratory failure. Reports describing the management of OI complicated by severe pneumonia in resource‐limited settings remain scarce.

**Case Presentation:**

We report a 13‐year‐old male with genetically unconfirmed but clinically evident OI who presented with high‐grade fever, productive cough, cyanosis, and severe respiratory distress. Chest X‐ray demonstrated right‐sided pneumonia and mild thoracic scoliosis. Airway evaluation showed no cervical spine instability or dentinogenesis imperfecta, and intubation was achieved without difficulty using a cuffed 6.5‐mm tube. Mechanical ventilation provided stabilization, with arterial blood gases normalizing. A comprehensive infectious workup was negative. The child improved with intravenous antibiotics, supportive care, and extubation on Day 3.

**Discussion:**

Respiratory compromise in OI arises from both skeletal and intrinsic airway abnormalities, including altered collagen architecture, bronchial wall thickening, and reduced chest‐wall compliance. Mild scoliosis and impaired airway clearance may have contributed to respiratory decompensation in this case. While BiPAP/CPAP may benefit selected OI patients, their use must be individualized; in this patient, noninvasive ventilation was not indicated due to discomfort risk and adequate response to invasive ventilation. Early screening for sleep‐disordered breathing and pulmonary dysfunction is recommended in OI.

**Conclusion:**

This case highlights the complexity of managing pneumonia in OI within resource‐limited contexts and underscores the need for multidisciplinary, individualized respiratory care.

## 1. Introduction

Osteogenesis imperfecta (OI) is a rare genetic disorder characterized by increased bone fragility, diminished bone mineral density, and a predisposition to fractures and skeletal deformities. The condition primarily stems from autosomal dominant mutations in genes responsible for Type I collagen synthesis and regulation, resulting in a heterogeneous clinical phenotype. In addition to skeletal manifestations, OI may present with extra‐skeletal features, including blue sclerae, dentinogenesis imperfecta, hearing loss due to otosclerosis, and hyperlaxity of joints [1]. It has an estimated birth prevalence of approximately 1 in 15,000 individuals [2]. Historically, the Sillence classification system categorized the disorder into four distinct types based on clinical presentation and radiographic findings [3]. To date, mutations in 20 distinct genes have been associated with the pathogenesis of OI [2, 4].

Respiratory manifestations commonly occur in OI patients and appear to significantly impact their health‐related quality of life. Notably, respiratory failure represents a potentially fatal complication that demonstrates higher prevalence in the OI population compared to unaffected individuals [5]. While pulmonary manifestations in OI have traditionally been attributed to secondary effects of scoliosis and vertebral compression fractures, emerging evidence indicates that respiratory dysfunction in OI patients may also involve an obstructive pathophysiology likely due to bronchial wall thickening [6].

To this date, there exists no report mentioning the management of OI with pneumonia in a resource‐limited setting, making this report utmost important for providing clinical insight into this case.

## 2. Case Report

A 13‐year‐old male patient was brought to the hospital by his mother, who reported a 3‐day history of fever, cough, and runny nose, with symptoms worsening over the last day to include fast breathing and chest indrawing. The fever was described as high‐grade (102 F), without chills, rigors, or diurnal variation. The cough was nonproductive initially but became productive with 2–3 bouts of cough per day by the third day of illness, leading to cyanosis and noisy breathing. There was no history of recent trauma, vomiting, headache, chest pain, or abnormal movements. Airway assessment revealed no cervical spine anomalies or restricted neck mobility. Dentinogenesis imperfecta was not observed. Mask ventilation was easy, and endotracheal intubation was achieved without difficulty using a cuffed 6.5‐mm endotracheal tube, which provided an adequate seal and ventilation. The patient was intubated and kept under mechanical ventilation due to respiratory distress and cyanosis. Mechanical ventilation was initiated using lung‐protective ventilation strategies with age‐appropriate tidal volumes and positive end‐expiratory pressure. FiO_2_ was adjusted to maintain oxygen saturation above 92%. The patient showed steady improvement in oxygenation and a reduction in work of breathing over the subsequent 48 h, permitting gradual weaning and successful extubation on Day 3. Noninvasive ventilation (NIV) was considered; however, due to severe hypoxemia, significant respiratory distress, and the need for airway protection, invasive mechanical ventilation was deemed more appropriate. Additionally, concerns regarding facial pressure intolerance and potential chest‐wall fragility in OI further supported the decision to proceed directly with controlled ventilation. He was started on intravenous (IV) piperacillin–tazobactam and vancomycin. Given the severity of presentation with hypoxemia, respiratory distress requiring mechanical ventilation, and radiographic consolidation, broad‐spectrum IV antibiotics were initiated empirically to cover severe community‐acquired pneumonia and potential resistant organisms. Piperacillin–tazobactam was selected for extended Gram‐negative coverage, while vancomycin was added to ensure coverage for methicillin‐resistant *Staphylococcus aureus*. Blood and urine cultures remained sterile. Despite negative cultures, IV antibiotics were continued for 7 days in view of the severity of illness and the initial requirement for ventilatory support. De‐escalation was not performed, as the patient demonstrated steady clinical improvement and completed the planned IV course prior to discharge. Cyanosis improved on the second day, and he was extubated on the third day following improvement in spontaneous respiratory effort, work of breathing, and decreasing oxygen requirement. The patient was born via spontaneous vaginal delivery at term, with a history of meconium aspiration and cried at birth. He experienced a first‐degree fracture at 2 months of age. There was no history of prior neonatal intensive care unit admission or postnatal events. The patient had a history of multiple fractures (8–9 times) in the bilateral upper limbs, with a radioulnar fracture at 3 months and a left tibia–fibula fracture at 6 years of age. Developmental history revealed delays: the patient played in groups but sat in a tripod position, with immature fine motor skills i.e., immature pincer grasp. Speech development was also delayed, with the patient able to say words and short stories. Immunizations were up‐to‐date as per the Expanded Program on Immunization (EPI) schedule, with gross motor skills reported as unable to achieve certain milestones.

On examination, the patient was alert and oriented, with bluish discoloration of the body, loss of consciousness, or abnormal movements. Vital signs were as follows:

Temperature: 102°F, heart rate: 130 beats per minute, respiratory rate: 50 breaths per minute, SpO2: 83% on room air. Chest examination revealed bilateral rhonchi with decreased air entry on the right side and crepitations in the right upper zone. Physical examination did not reveal any cardiac and abdominal abnormalities, and there were no positive central nervous system findings.

There were no symptoms suggestive of urinary tract infection and diarrhea.

**FIGURE 1:**
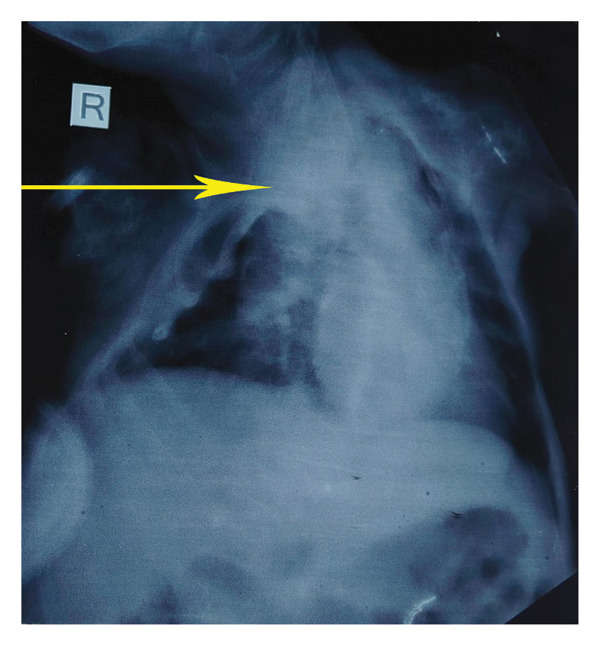
Chest X‐ray: yellow arrow showing the area of opacity and mild thoracic scoliosis, a known skeletal manifestation of osteogenesis imperfecta.

Impression: Severe pneumonia with OI in a child, which was confirmed via chest X‐ray AP view as demonstrated in Figure 1.

**FIGURE 2:**
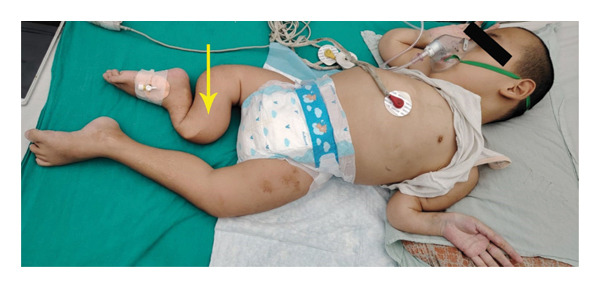
Yellow arrow showing anterolateral bowing deformity of the deformed limb due to recurrent fractures.

However, the type and genetic mutation could not be identified due to resource and financial limitations of the setting. Figure 2 demonstrates anterolateral bowing deformity of the lower limb, likely resulting from malunited fractures secondary to OI.

A comprehensive infectious workup, including malaria antigen testing, scrub typhus RDT, rK39 for leishmaniasis, *Leptospira* serology, dengue panel, blood culture, and urine culture, was negative, effectively ruling out common infectious causes of fever as detailed in Table 1.

**TABLE 1:** All the investigations to rule out causes of fever.

Category	Test	Result	Unit	Reference range (normal values)
Hematology	Hemoglobin	14.6	gm%	11–16
	PCV	44.6	%	36–52
	Total leukocyte count	5830	cell/cmm	4000–11,000
	Neutrophil	75.9	%	40–75
	Lymphocyte	12.7	%	20–45
	Monocyte	11.1	%	2–10
	Eosinophil	0.3	%	1–6
	Basophil	0	%	0‐1
	Platelet count	297,000	cell/cmm	150,000–4,00,000
	Mean corpuscular volume (MCV)	91.3	fl	80–96
	Mean corpuscular hemoglobin (MCH)	29.9	pg	27–34
	Mean corpuscular hemoglobin concentration (MCHC)	32.7	gm/dL	32–36
	Mean platelet volume (MPV)	8.2	fl	6.5–12.0

Biochemistry	Sodium	139.6	mmol/L	135–146
	Urea	12.5	mg/dL	15–40
	Creatinine	0.12	mg/dL	0.35–0.86
	Potassium	3.2	mmol/L	3.5–5.0
	Total calcium	8.8	mg/dL	8.5–10.5
	Phosphorus	2.5	mg/dL	2.5–4.5
	Alkaline phosphatase (ALP)	118.07	U/L	35–130

Serology	Dengue serology NS1	Negative	—	Negative
	IgM	Negative	—	Negative
	IgG	Negative	—	Negative
	Leptospirosis IgG	Negative	—	Negative
	Malaria rapid antigen test	Negative	—	Negative

Microbiology	Scrub typhus RDT	Negative	—	Negative
	rK39 test	Negative	—	Negative
	Blood culture and sensitivity	Culture sterile after 48 h of aerobic incubation at 37°C	—	No growth (sterile)
	Urine culture and sensitivity	Culture sterile after 24 h of aerobic incubation at 37°C	—	No growth (sterile)

Urine routine examination (RE/HE)	Sugar	Negative	—	Negative
	Protein	Negative	—	Negative
	WBC	4–6	/HPF	0–5
	Epithelial cells	4–6	/HPF	0–5
	Granular cast	Seen	—	Not typically present
	Bacteria	Seen	—	Not typically present

Malaria test	*Plasmodium vivax*/*Plasmodium falciparum* for malaria	Not seen	—	Not seen

Investigation findings of the patient were as follows, as demonstrated in Table 1

## 3. Discussion

While the majority of existing research primarily addresses the pediatric manifestations of OI, this condition persists as a chronic disorder with substantial morbidity that continues to impact individuals throughout their adult lives. Effective management of OI necessitates a multidisciplinary approach. Pharmacological interventions aim to enhance bone mineral density, mitigate fracture risk, and alleviate pain. With the exception of the most severely affected individuals, patients generally derive benefits from engaging in a consistent exercise regimen and maintaining an active lifestyle [7]. Occupational therapists play a critical role by facilitating lifestyle adaptations, which include the provision of assistive devices to support daily activities and mobility.

Pharmacotherapeutic research primarily focuses on promoting growth and enhancing bone mineralization. Initial findings from a study investigating the application of growth hormones provide a rationale for further clinical trials in children diagnosed with Type IV OI [8]. More contemporary research indicates that recombinant human growth hormones exert an anabolic effect on bone tissue; however, they do not significantly enhance bone density or strength. Numerous studies advocate for the use of IV pamidronate, with evidence demonstrating a reduction in the incidence of new fractures, an increase in bone mineral density, and improvements in mobility and ambulation. Additionally, two studies on pamidronate have reported significant alleviation of chronic pain and fatigue in affected individuals [9].

Orthopedic management is initiated with the identification of fractures. During the neonatal period, physicians may opt for splinting fractures rather than applying casts, as casting can initiate a detrimental cycle wherein immobility exacerbates osteoporosis, thereby increasing the risk of subsequent fractures. In more severe cases, hip spica casts may be employed for lower limb fractures, offering greater stability and reduced pressure on the affected area compared to conventional casts. As the child progresses to school age, orthopedic interventions may involve surgical procedures such as the insertion of intramedullary rods to enhance support for ambulation and to address skeletal deformities. Spinal fusion may be required in instances of progressive scoliosis [10]. Additionally, bracing devices can facilitate ambulation as the child develops.

Providing emotional support constitutes a critical component of nursing care. Upon receiving a diagnosis of OI, parents often experience grief over the perceived loss of an idealized, healthy child. Familiarity with the stages of grief equips nurses to better comprehend and address the emotional needs of parents during this challenging period. Parents require opportunities to express their emotions, as the realization that routine actions such as holding their child may result in fractures and increased pain can engender significant fear. To effectively nurture and care for their child, parents must navigate through their grief and anxiety. Early education on safe handling techniques enables parents to confidently hold their child, fostering bonding and attachment [1]. Furthermore, parents benefit from guidance on medication administration as well as the proper management of casts and splints.

The correlation between OI and hospital admissions due to respiratory conditions was more pronounced among female patients compared to male patients with OI, although the precise etiological factors remain unidentified. It may be hypothesized that this observed disparity could be partially attributed to anatomical and physiological distinctions, including reduced lung volumes and diminished respiratory muscle strength in female patients with OI, which may predispose them to a higher incidence of respiratory complications [5].

The management of OI complicated by pneumonia presents significant challenges, primarily due to the impaired ability of affected children to effectively clear sputum, coupled with an elevated and recurrent risk of respiratory infections. One study suggests that Type I collagen composes nearly 75% of the myocardium and is essential to vascular structure, alveolar support, and bronchial wall stability. Defects in this collagen, therefore, contribute directly to cardiopulmonary impairment in OI, beyond the effects of skeletal deformities [6].

### 3.1. Respiratory Complications in OI

Respiratory morbidity in OI arises from both skeletal and intrinsic pulmonary abnormalities. Mild thoracic scoliosis, as seen in our patient, can reduce chest‐wall compliance and contribute to a restrictive ventilatory defect. Beyond these skeletal changes, emerging evidence suggests that intrinsic airway abnormalities including altered collagen architecture, thickened bronchial walls, and reduced alveolar support directly impair respiratory function in OI patients. Heightened susceptibility to pulmonary complications is attributable to these combined skeletal and airway factors, which predispose children with OI to recurrent infections and impaired secretion clearance.

### 3.2. Sleep‐Related Breathing and Pulmonary Monitoring

Children with OI also demonstrate increased rates of sleep‐disordered breathing and obstructive sleep apnea. Although polysomnography and spirometric assessment could not be performed during the acute illness, targeted respiratory screening is recommended once clinically stable. Clinicians should consistently assess OI patients, especially those with impaired ambulation, high body mass index, severe trunk deformities, or progressive scoliosis, for evolving pulmonary dysfunction. Early spirometry is particularly important in Type III OI, where restrictive physiology is common.

### 3.3. NIV Considerations

Bilevel positive airway pressure (BiPAP) and continuous positive airway pressure (CPAP) can be beneficial in selected OI patients; however, their use must be individualized. Significant chest‐wall fragility, facial sensitivity, or risk of pressure‐related harm may limit tolerance. In our patient, the nature of respiratory failure and adequate response to invasive ventilation made NIV unnecessary. Arterial blood gases remained within normal limits during mechanical ventilation, indicating that the acute decompensation was primarily due to pneumonia superimposed on the underlying restrictive physiology of OI.

## 4. Airway Management Considerations in OI

Airway management in OI is often challenging due to potential cervical spine instability, limited neck mobility, midface hypoplasia, and dentinogenesis imperfecta. These anatomical features may complicate mask ventilation and tracheal intubation. In this case, no cervical spine abnormalities or dental fragility were present, and intubation was successfully performed with a cuffed 6.5‐mm endotracheal tube. This underscores the importance of thorough preintubation airway assessment, as anatomical variability can significantly influence airway strategy and ventilation planning in children with OI.

We recommend initiating spirometric assessment early in Type III OI and in patients with marked scoliosis to anticipate the commonly encountered respiratory complications [11]. Another study revealed that clinicians should consistently assess OI patients, especially those with impaired ambulation, high body mass index, trunk deformities, or severe forms, for signs of sleep‐disordered breathing [1]. While otorhinolaryngologist review is essential, in cases without upper‐airway abnormalities, polysomnography and targeted treatment are recommended [12]. BiPAP and CPAP may benefit selected OI patients but may be unsuitable in those with significant chest‐wall fragility or facial discomfort, as noted in recent evidence [4]. In our case, CPAP/BiPAP was not indicated due to the nature of respiratory failure and the risk of pressure‐related harm.

This case underscores the inherent complexities in treating such patients and emphasizes the critical need for a comprehensive, multidisciplinary approach to care. This approach should encompass not only acute medical interventions but also long‐term rehabilitation strategies, including consistent physiotherapy to enhance respiratory function and facilitate sputum clearance. Furthermore, the provision of sustained physical and emotional support is essential to address the multifaceted needs of these children, enabling them to achieve a more manageable and fulfilling lifestyle despite the chronic nature of their condition. Such support is vital for mitigating the physical limitations imposed by OI and for fostering resilience against the recurrent respiratory challenges that these patients face, ultimately improving their overall quality of life.

## 5. Conclusion

OI presents unique challenges when complicated by acute respiratory illness, particularly in resource‐constrained environments. This case illustrates how skeletal deformities, intrinsic airway abnormalities, and impaired secretion clearance can predispose OI patients to severe pneumonia and respiratory failure. Early airway evaluation enabled safe intubation, and prompt initiation of mechanical ventilation facilitated clinical stabilization. Although NIV may be beneficial in selected OI cases, its use must be carefully individualized, as demonstrated by its limited suitability in our patient. Comprehensive multidisciplinary care including vigilant respiratory monitoring, physiotherapy, and caregiver support is essential to improving outcomes. Strengthening diagnostic capability and access to pulmonary evaluation may enhance future management of similar cases.

## Funding

There are no sources of funding.

## Ethics Statement

Ethical approval is not required for case reports.

## Consent

Written informed consent was obtained from the patient’s legal guardian for publication of this case report and accompanying clinical information. A copy of the signed consent form is available for review by the editor‐in‐chief of this journal on request.

## Conflicts of Interest

The authors declare no conflicts of interest.

## Data Availability

Data sharing is not applicable to this article as no datasets were generated or analyzed during the current study.
